# Supplementary material for: Segregation between the parietal memory network and the default mode network: effects of spatial smoothing and model order in ICA
Source: Sci Bull (Beijing). 2016 Dec 5;61(24):1844–54. doi: 10.1007/s11434-016-1202-z (PMC5167777; doi:10.1007/s11434-016-1202-z)
Supplement: Supplementary file 1 — Supplementary material 1 (DOCX 6954 kb) [file 11434_2016_1202_MOESM1_ESM.docx]

**Supplementary Material**

Segregation between the parietal memory network and the default mode network: Effects of spatial smoothing and model order in ICA

Yang Hu^1,2^, Jijun Wang^3^, Chunbo Li^3^, Yin-Shan Wang^1,2^, Zhi Yang^1,3^*, Xi-Nian Zuo^1^


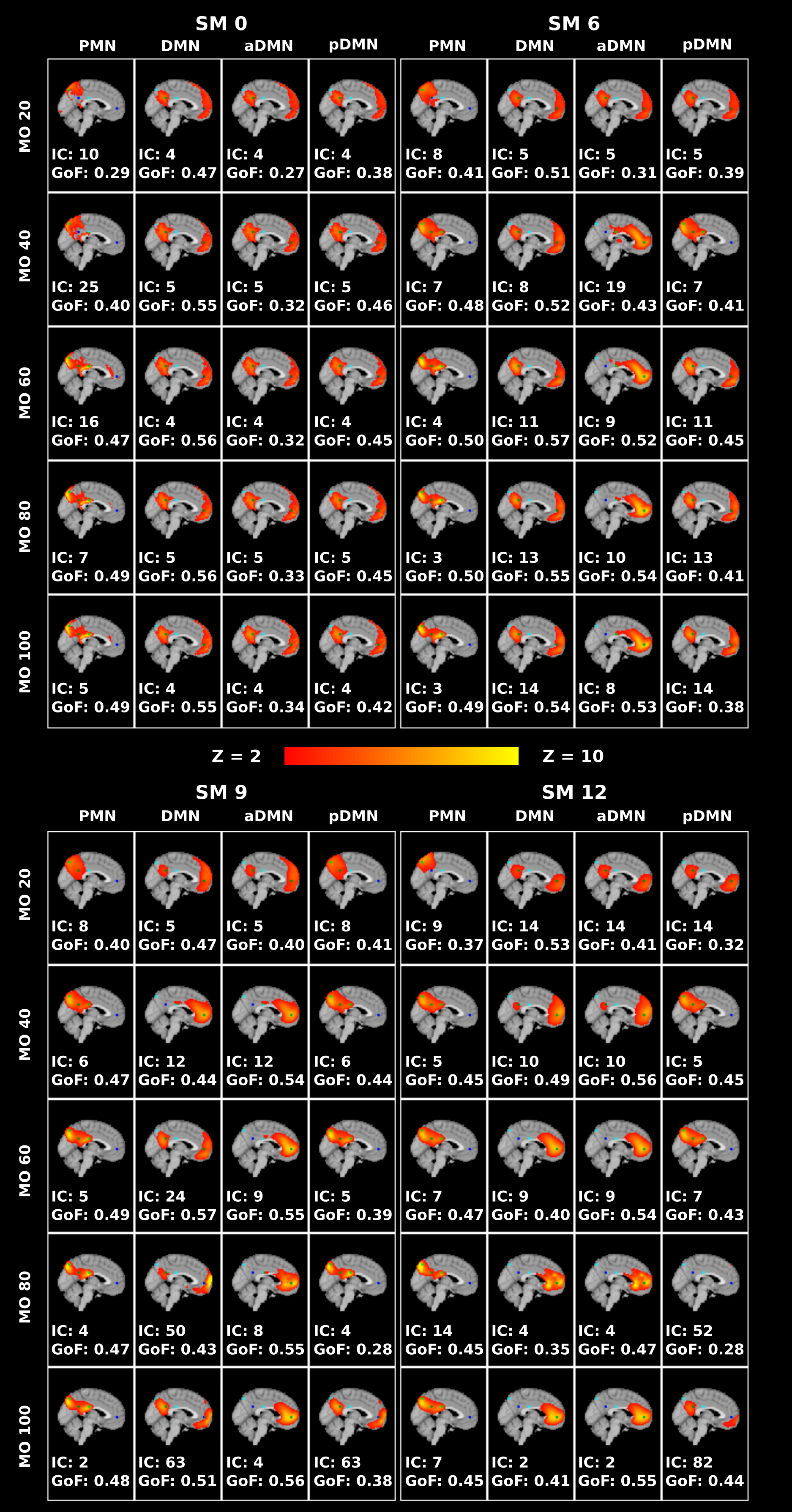


Figure S1. The gRAICAR component maps representing PMN, DMN, aDMN, and pDMN were selected by the highest correlation with templates in different combinations of model orders and spatial smoothing sizes. The maps were thresholded at Z > 2 and overlaid on a MNI anatomical template for visualization, of which the index and correlation with template were listed below.


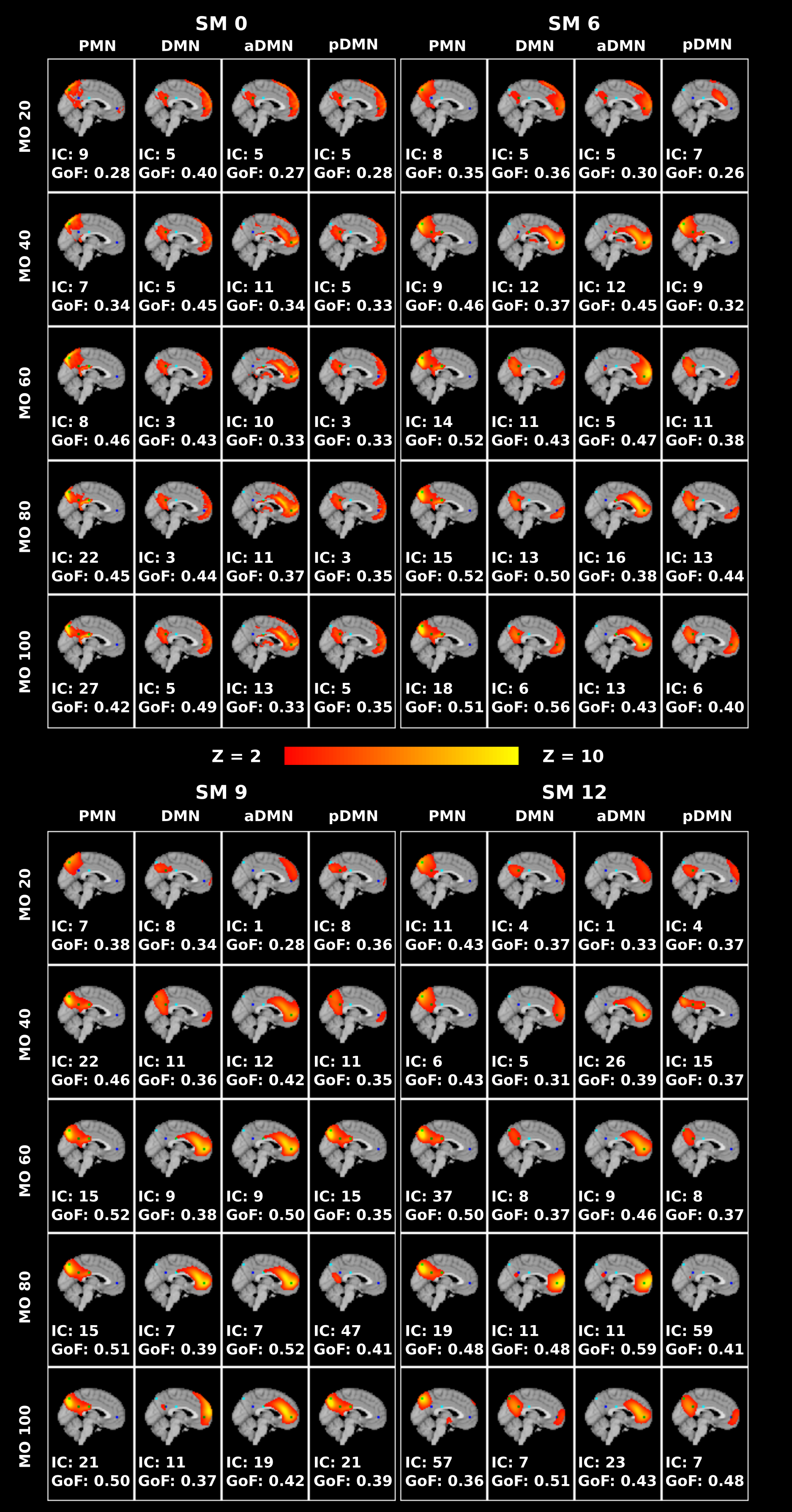


Figure S2. The IVA-GL component maps representing PMN, DMN, aDMN, and pDMN were selected by the highest correlation with templates in different combinations of model orders and spatial smoothing sizes. The component maps were thresholded at Z > 2 and overlaid on a MNI anatomical template for visualization, of which the index and correlation with template were listed below.


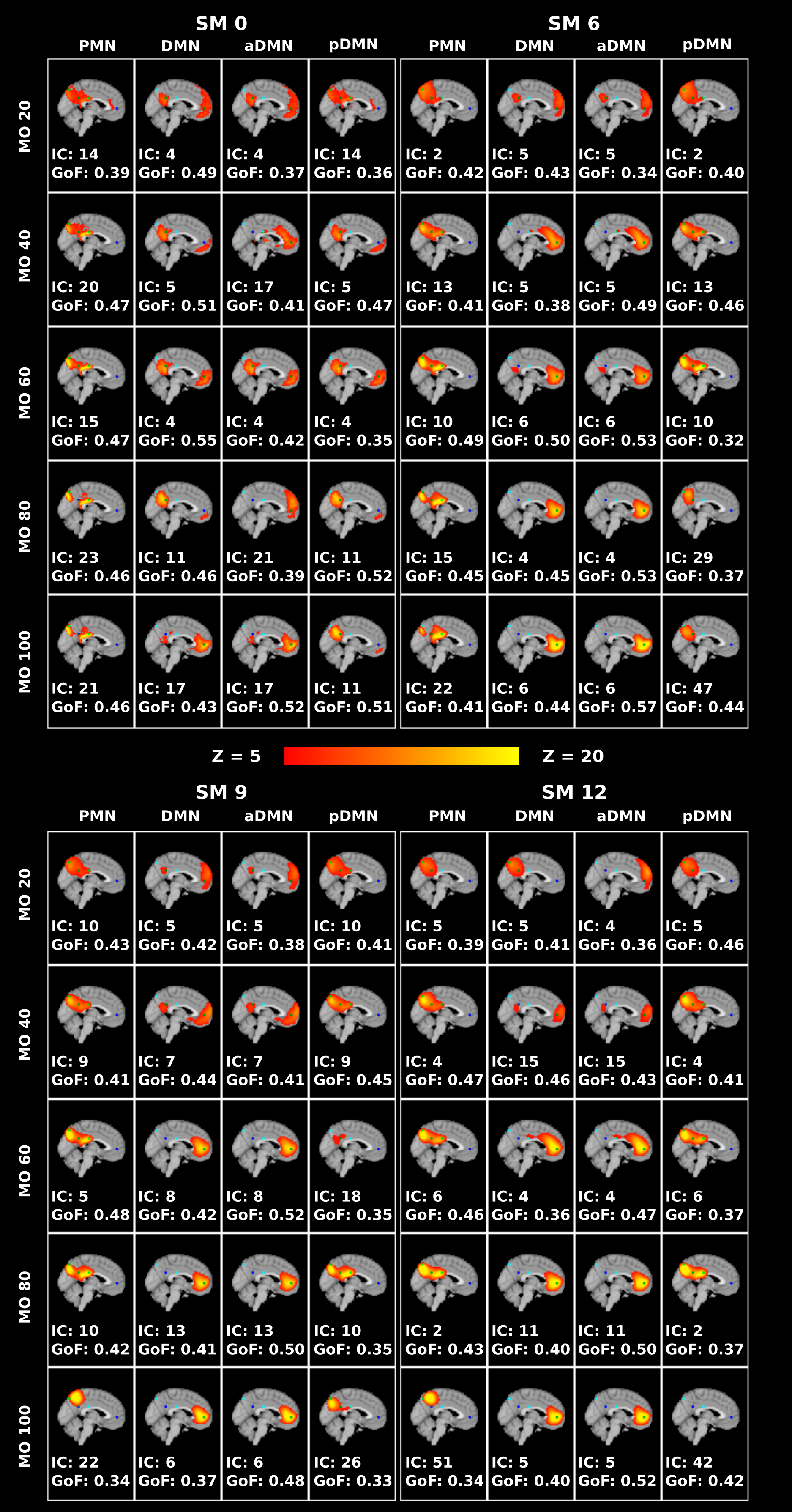


Figure S3. The TCgICA component maps representing PMN, DMN, aDMN and pDMN were selected by the highest correlation with templates in different combinations of model orders and spatial smoothing sizes. The component maps were thresholded at Z > 5 and overlaid on a MNI anatomical template for visualization, of which the index and correlation with template were listed below.


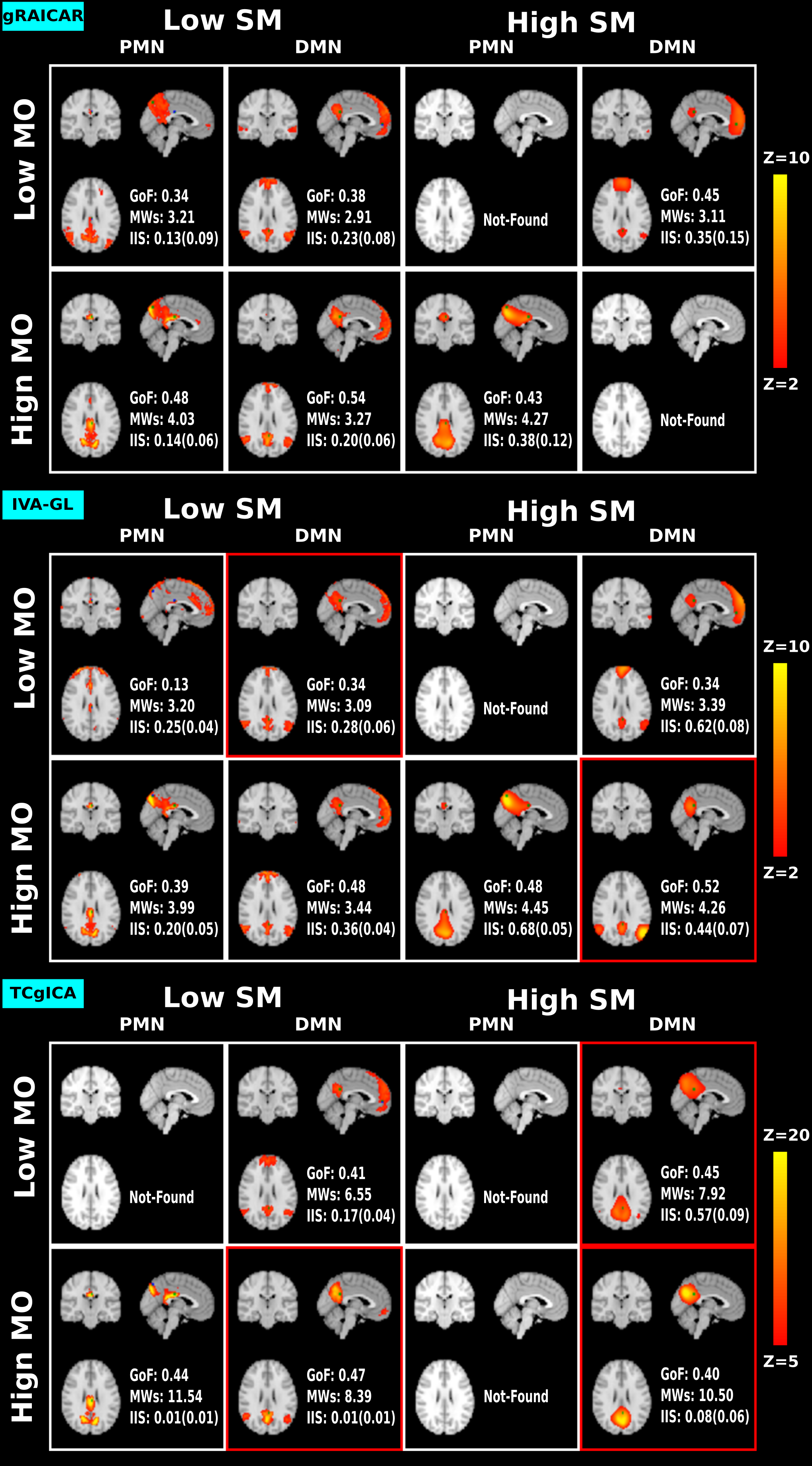


Figure S4. The selected IC maps representing PMN and DMN in four combinations of spatial smoothing levels and model orders across three algorithms in a sub-sample with narrower age range (20-30, *n*= 29). A red border means that the RSN is identified as posterior DMN.
